# Determinants of delayed tuberculosis treatment among patients in Selangor: A study protocol

**DOI:** 10.1371/journal.pone.0266746

**Published:** 2022-04-25

**Authors:** Punitha Makeswaran, Shamsul Azhar Shah, Nazarudin Safian, Nor Asiah Muhamad, Abdul Aziz Harith

**Affiliations:** 1 Department of Community Medicine, UKM Medical Centre, Cheras, Kuala Lumpur, Malaysia; 2 Evidence Based Healthcare Medicine Sector, National Institutes of Health, Ministry of Health, Shah Alam, Selangor, Malaysia; 3 Occupational Health Research Centre, Institute for Public Health, Ministry of Health Malaysia, Shah Alam, Selangor, Malaysia; Indian Institute of Technology Delhi, INDIA

## Abstract

The high prevalence rate and ever-increasing incidence of tuberculosis (TB) worldwide remain a significant issue in healthcare. In Malaysia, the incidence and mortality rate of TB is increasing due to the delayed presentation of TB patients to healthcare facilities. However, there is a lack of local studies on the contributing factors of delayed presentation of TB patients in Malaysia. This study aims to establish a social epidemiology framework by analysing social factors including socio-epidemiological, socio-cultural, and health-seeking behaviours associated with the delay in seeking TB treatment among patients in Selangor, Malaysia. A sequential exploratory mixed-method study design that combines qualitative and quantitative research methods will be employed. This study will recruit adult patients who have been diagnosed with TB using chest X-ray and sputum smear microscopy. Four districts with the highest recorded cases in the state of Selangor will be selected as study locations. The qualitative study will involve a Focus Group Discussion (FGD) to explore six components, namely demographic, socio-cultural, health-seeking behaviours, social support and resources, previous knowledge and experience with illness, and treatment pathway. Meanwhile, the quantitative study will incorporate a structured survey that will be developed based on the themes identified in the qualitative phase and a review of several studies in the literature. Several quality control measures will be taken while ensuring that the survey questionnaires are anonymised and participants’ confidentiality is maintained. The data obtained from both qualitative and quantitative approaches will be combined to provide a more robust assessment of the study. Given that this study will focus on districts with high recorded cases of TB in Selangor, the findings might assist to address TB-related issues such as the increasing incidence and mortality rates, which are mainly attributed to the delayed presentation of TB patients to healthcare facilities.

## Introduction

Tuberculosis (TB) is an airborne communicable disease caused by Mycobacterium tuberculosis (MTB), an anaerobic pathogenic bacillus [1, 2]. It is recognised as one of the infectious diseases with a high mortality rate that spreads through airborne transmission. About one-fourth of the world’s population is estimated to be infected with MTB [3]. Although the vast majority are non-infectious and asymptomatic, they are still at risk of developing active TB disease and becoming infectious. The manifestations of TB disease differ among the population, some patients develop symptoms within weeks of being infected before their immune system can fight the bacteria, whereas some may fall sick only years later when their immune system becomes weakened for other reasons [4].

As an airborne disease, TB spreads through infectious droplets known as droplet nuclei that can be easily transmitted to other hosts or remain in the air for several hours, depending on the surrounding environment [4, 5]. The transmission usually occurs when an individual inhales the droplet nuclei and the bacterium attacks the lungs. Additionally, the infection can affect other organs such as the kidney, brain, and spine. Common TB symptoms include chest pains, loss of appetite, loss of weight, fatigue, fever, muscle and joint weakness. The patients also suffer from prolonged cough for more than 3 weeks, with or without haemoptysis [4, 5].

Due to the high incidence of TB, many developed and developing countries worldwide are facing a heavy healthcare burden in managing this disease, including India, Indonesia, and Nigeria [6]. Based on the STOP TB strategy by The World Health Organisation (WHO), the global prevalence and mortality rate of TB by 2015 should reduce by 50% compared to that reported in 1990 [4]. In 2019, an estimated 10 million cases, or equivalent to 133 cases per 100,000 populations of TB was reported globally [7]. Moreover, in 2019, the number of new cases of TB globally was 7.1 million, an increase from 7 million in 2018 and 6.4 million in 2017, as well as a marked increase of 5.7 to 5.8 million annually between 2009 and 2012 [2, 7, 8]. Out of the new cases in 2019, 62% were reported in Southeast Asia (SEA) and the Western Pacific areas, followed by 25% in the African region. In the SEA, the incidence of TB was 226 cases per 100,000 population [9]. Concurrently, the number of TB-related death was 2.8 per 100,000 populations, with the highest incidence recorded in the Philippines (555 cases per 100,000 population) and the lowest in Malaysia (93 cases per 100,000 population) (WHO, 2018).

TB accounts for the second-highest mortality from a single infectious agent after HIV/AIDS. The aetiology of TB is complex. The risk of contracting TB is higher among people with existing health conditions, especially among patients with Human Immunodeficiency Virus (HIV). Specifically, they are 30 times at higher risk of developing TB than those without HIV [10]. Furthermore, TB is classified as the leading cause of death among HIV patients due to the increased risk for inflammatory immune reconstitution (IRIS) [10]. In 2017, 9% of TB cases were detected among persons with HIV infection [2]. Besides, people with underlying diabetes mellitus are also at a higher risk of developing TB. A recent study reported that diabetic patients are three times more likely to develop TB [11]. The incidence of TB patients with diabetes mellitus is particularly high in the low- and middle-income countries (LMIC). In addition, another study found that men with adenocarcinoma as well as women with asthma or early-stage adenocarcinoma suffered from an increased risk of TB-related mortality [12].

Apart from existing medical conditions, socio-economic and cultural factors are also the determinants of TB infection. Poor economic status can limit the economic resources of individuals, causing them to live in an overcrowded housing area with a cheaper rental rate. Past evidence highlighted that an overcrowded living condition can predispose to an increased risk of developing TB among the dwellers [13]. In addition, poor economic status also reflects a lower education level that impedes the understanding of TB, especially in terms of the transmission process and symptoms of TB. Previously published studies have stressed the importance of reducing education inequalities to bring down the incidence of TB [14, 15].

In addition, TB is commonly known as the barometer of social welfare. In other words, the prevalence is a reflection of the effectiveness of the social welfare programme offered by the government to the citizens. A high number of TB cases in certain populations may indicate poor urban planning and healthcare provision by the government [16]. Other social factors associated with TB infection include quality of life, population growth, malnutrition, lifestyle habits such as smoking and alcohol consumption, overcrowded family homes, early marriages, poor knowledge, and lack of awareness on the cause and transmission of TB [17]. These factors are also correlated in the occurrence and transmission of TB.

The increasing trend of global TB infection in recent years could also be attributed partly to the improved diagnostic method of the disease. Detection of the infectious status and the stage of disease in TB patients is crucial as it influences the prognosis and treatment outcomes of TB cure. Delayed presentation of TB is associated with a higher failure rate of treatment and death [18]. Moreover, in clinical practice, the diagnosis of TB can be challenging as it closely mimics the symptoms of pneumonia. Very often, patients are treated as pneumonia and prescribed antibiotics before TB is officially diagnosed [18]. On a similar note, it is also difficult to diagnose individuals with TB/HIV co-infection [19]. Many of the current diagnostic techniques lack precision in detecting positive cases. They can also be time-consuming, costly, or require advanced laboratory equipment that is limited in developing countries [19].

The TB diagnostic tools endorsed by WHO can be divided into traditional tools such as symptom screening, chest x-ray, microscopy staining, or newer tools such as Interferon-gamma Release Assay (IGRA), Fluorescent Staining (LED), Liquid Media Culture, and Polymerase Chain Reaction [20]. The newer tools significantly improve the case detection rate, based on the declining mortality rate by 42% and a higher rate of treatment success (82%) among newly diagnosed cases between 2000 and 2017 [2]. Sputum is the gold standard for TB diagnosis. However, some difficulties in obtaining the sputum samples have been reported, especially among children and HIV-infected individuals [19].

Diagnosing and treating TB at a late stage may negatively impact the effectiveness of the treatment plan. Therefore, it is imperative to address factors that contribute to the delayed presentation among TB patients, such as social factors, lack of awareness and knowledge of TB, healthcare provider-related barriers, and belief in traditional treatment.

### Why is it important to conduct this study?

In recent years, the incidence and mortality rates of TB is increasing rapidly in Malaysia with the influx of immigrants. A high incidence of TB can negatively impact the country’s social development and offset the economic growth in various aspects. To reduce the incidence of TB and its mortality rate, suspected patients must be diagnosed at the earliest stage. However, due to a lack of knowledge and awareness of TB as well as limited healthcare facilities, patients with TB are often diagnosed late.

In Malaysia, Selangor recorded a total of 375 deaths and the highest number of TB cases in 2018 (5,071 cases), contributing to almost 18% of the overall cases in the country [21]. Selangor is the most populated state in Malaysia with a population of more than 6.4 million. It comprises nine districts and the four bigger districts namely Hulu Langat, Gombak, Petaling, and Klang accounted for the highest percentages of TB cases in Selangor [21]. Currently, there is a lack of evidence on the contributing factors of delayed treatment among TB patients. Therefore, this protocol outlines a study that is planned to explore the reasons behind the delay in seeking TB treatment among TB patients in Malaysia.

## Objectives

To develop a framework by analysing the social factors associated with delay in seeking treatment among TB patients in Selangor. This study will describe the socio-epidemiological characteristics (socio-demographic and socio-cultural) of TB patients in Selangor, Malaysia. This study will determine the common barriers faced by health providers in Selangor and the association between patients’ health-seeking behaviour and their social epidemiological factors (socio-demographics and socio-cultural), as well as health provider barriers. Finally, this study protocol aims to construct a predictive social epidemiology framework to identify contributing factors towards the delay in seeking treatment among TB patients in Selangor, Malaysia.

## Research questions

Due to the high incidence of TB infections in the state of Selangor, the research aims to answer the following questions:

1. What is the incidence rate of TB in Selangor?
2. What is the distribution of socio-demographic factors among the TB patients in Selangor?
3. What is the distribution of socio-cultural factors among the TB patients in Selangor?
4. What is the distribution of health-seeking behaviours among the TB patients in Selangor?
5. What are the barriers faced by healthcare providers that can influence the TB diagnosis?
6. How do the socio-demographic, socio-cultural, and health provider factors interact to influence health-seeking behaviours among TB patients?

To answer the aforementioned research questions, this research focuses on the identification of risk factors for delayed presentation of TB in Selangor.

## Methods

### Study design and study area

This study will adopt a mixed-method research design. We will combine quantitative and qualitative approaches to obtain comprehensive results regarding the research questions [22]. The exploratory sequential mixed methods design will be conducted for the data collection. Data collection will be performed among patients who have been diagnosed with TB using chest X-ray and sputum smear microscopy. Four districts with the highest recorded cases in the state of Selangor will be selected as study locations. The sampling frame will be determined from the MyTB system database and patients fulfilling the inclusion criteria will be identified as sampling units. Thereafter, they will be categorised based on the four districts to ensure there are respondents from each district.

### Study population

The study population will comprise TB patients in the state of Selangor.

### Inclusion criteria

The following inclusion criteria will be used in recruiting the study participants:

- Confirmed TB patients that were diagnosed using chest X-ray and sputum smear microscopy.
- TB patients from districts with the highest recorded cases in Selangor
- Informed consent to participate in the study

### Exclusion criteria

The exclusion criteria are as follows:

- Suspected or unconfirmed TB patients.
- TB patients from districts with low recorded cases of TB in Selangor
- Decline to provide informed consent to participate in the study

### Sample size calculation and sampling technique

Sample size will be calculated using the formula for analytical and cohort studies [23, 24] as shown below:

$$n^{'}=\frac{{\left[z_{1-\alpha /2}\;\sqrt{2\overset{-}{P}\;\left(1-\overset{-}{P}\right)}+\;z_{1-\beta }\;\sqrt{P_{1}\left(1-P_{1}\right)+\;P_{2}\left(1-P_{2}\right)}\right]}^{2}}{({P_{1}-\;P_{2})}^{2}}$$

$$n=\frac{n'}{4}\;{\left(1+\;\sqrt{1+\;\frac{2\;(2)}{n^{'}\left(P_{2}-\;P_{1}\right)}}\;\right)}^{2}$$

Where,

n = Sample size

Z1 – α/2 = Z statistic for level of confidence of 95% = 1.96

Z1-β = Z statistic for 90% power = 1.28

P_1_ = Proportion with present condition in one group

P_2_ = Proportion with present condition in another group

P_1_ and P_2_ will be obtained from previous prevalence studies on patient and treatment delays among different groups of PTB patients. The obtained values will be substituted into the equation to compute the required sample size (n). Thereafter, the calculated sample size will be adjusted for eligibility by assuming that approximately 30% had delayed TB presentation [25]. Additionally, the sample size will be adjusted for the response rate.

Probability proportionate to size (PPS) technique will be used to allocate sample size for each district to enable proportionate allocation of patients from each healthcare facility in the district. A simple random sampling technique will be used to obtain the required number of patients from each district. A purposive sampling technique will be used for the selection of healthcare facilities in the district with a high prevalence of TB. Patients at each facility will be selected randomly using the MyTB database. Once the samples for the quantitative study have been identified, a subsample of respondents will be invited to join the qualitative study, with an equal number from each district.

### Qualitative phase: Study instrument and focus group discussion

Upon identifying the samples for the quantitative study, a subsample will be selected among them to participate in the Focus Group Discussion (FGD). As a rule of thumb, 10% of the estimated sample size for the quantitative study will be selected to join the FGD. Meanwhile, the number of participants participating in the FGD will be equally distributed across the four districts. A semi-structured questionnaire will be used for the FGD respondents to share their thoughts and opinions. The qualitative study aims at identifying and better understanding the social factors related to their delayed presentation to the healthcare centres.

Six components will be explored during the discussion, namely demographic, socio-cultural, and health-seeking behaviours, social support and resources, previous knowledge and experience with illness, as well as treatment pathway. The guide will be revised in subsequent FGD to probe any new emerging themes. In all the FGDs, the discussions will be audio-recorded and transcribed verbatim after that. The concept of theoretical saturation will be used to ensure no new conceptual information emerges from the discussion. Subsequently, all the findings from the qualitative phase will be transcribed and coded. The identification, coding, and categorisation of meaningful patterns into themes and subthemes will be conducted to facilitate the construction of a structured questionnaire for the quantitative study. Interview transcripts will be analysed using NVivo (Version 12).

### Quantitative phase: Instrument development

A structured questionnaire will be constructed based on the themes identified in the qualitative study and a review of several studies in the literature. Operational definitions will be developed based on the themes obtained. The questionnaire will undergo content validity by three field experts. Then, it will be distributed to ten healthcare professionals for face validity assessment. Necessary modifications will be made based on the comments received. The final questionnaire will then be translated into the local language, Bahasa Malaysia, and pre-tested on 30 TB patients from selected hospitals and health clinics for the reliability or internal consistency. Based on the field experts’ opinions and pilot testing, potential ceiling and floor effects from the questionnaire items that may affect accurate data interpretation will be prevented.

For the quantitative phase, a cross-sectional study using the developed questionnaire will be distributed to the patients identified through the PPS technique at all selected hospitals and health clinics in all four districts. Finally, the data obtained from both qualitative and quantitative approaches will be combined to provide a more robust assessment of the study. The qualitative findings from the viewpoints of the participants in FGD will facilitate the interpretation of the study results, whereas the quantitative results will provide a better understanding of the study objectives and research questions [22]. An expert panel will be engaged to construct the social epidemiology framework model for delayed presentation of TB.

**Fig 1:**
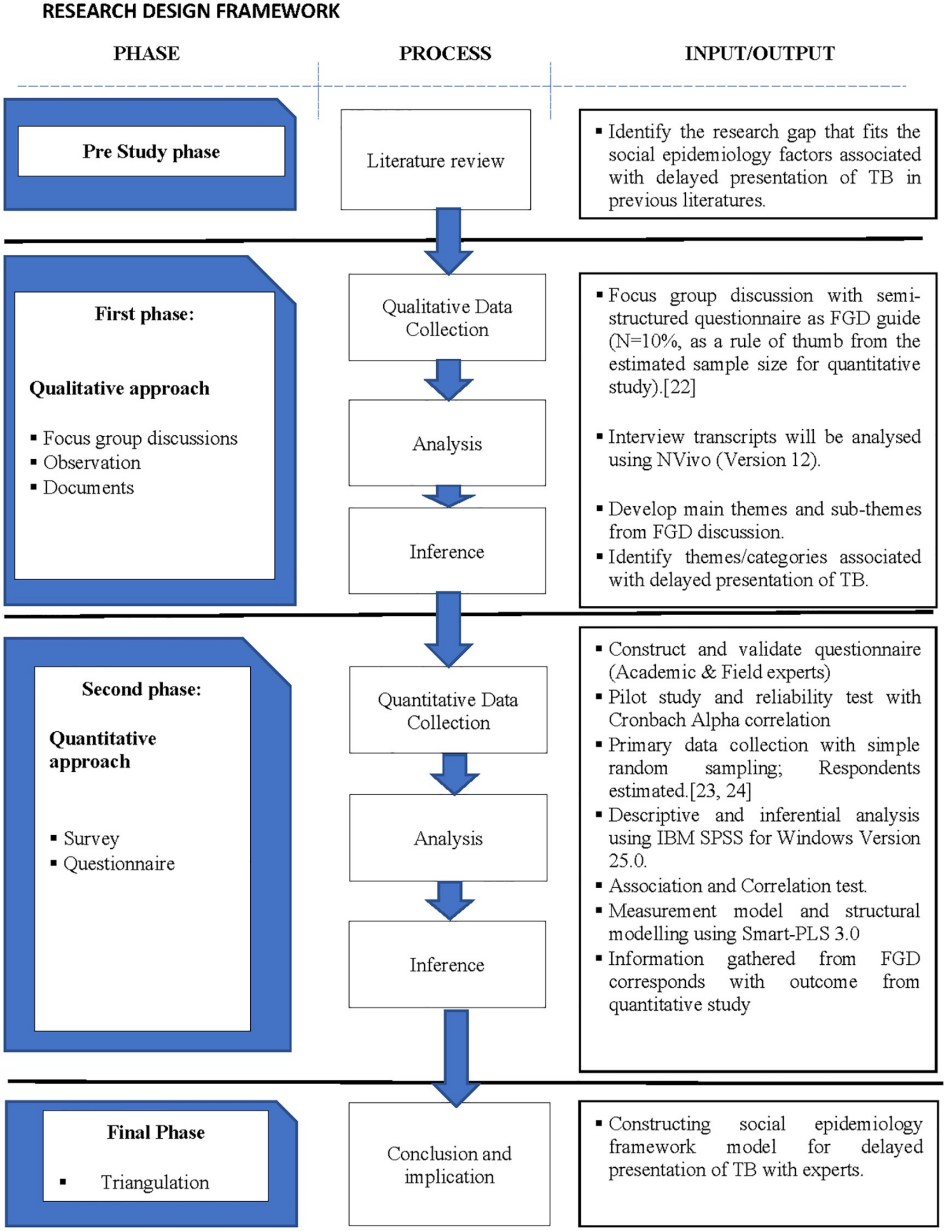
Research design of the different study phases.

The research design in this study offers the strengths of both quantitative and qualitative approaches, thus providing a more robust outcome for this study. A mixed-method research design can also broaden the dimension and scope of the study [22]. Table 1 shows the research matrix and Fig 1 presents the overall research design of this study according to each research question.

**Table 1:** Research matrix.

RESEARCH OBJECTIVES	QUANTITATIVE	QUALITATIVE
To describe the incidence of TB in Selangor and the health-seeking behaviour (timeline between the onset of symptoms to the first presentation to the healthcare facility and medical visit) among the patients.	√	√
To describe the social epidemiological characteristics (socio-demographic and socio-cultural) of the TB patients in Selangor.	√	
To determine the types of health provider barriers that are prevalent in Selangor.	√	√
To determine the association between health-seeking behaviour and social epidemiological factors (socio-demographics and socio-cultural) as well as health provider barriers.	√	
To investigate the key factors that contribute to delayed presentation of TB patients.		√
To construct a predictive social epidemiology framework for the identification of delay in seeking treatment among TB patients in Selangor.	√	√

### Ethical consideration

This study will involve two key ethical principles, namely honesty and respect for human rights [22]. Since adults with TB disease will be the study participants, certain ethical questions need to be addressed. The confidentiality and safety of respondents must be guaranteed. Therefore, informed consent will be obtained from the participants in survey questionnaires. The researcher shall provide important information and purpose about the study with a cover letter on the questionnaire to the participants before obtaining their informed consent. This study protocol has been approved by the Research Ethics Committee UKM (JEP-2020-589) and the Medical Research & Ethics Committee Ministry of Health Malaysia (NMRR-20-1595-55723).

### Management

The researchers will ensure the protection of participants’ anonymity. Meanwhile, data concerning people will be kept confidential to ensure that their identities and any form of identifying information are protected. A coding system will be employed to represent the clinical records, research instruments or any used documents containing participants’ data. Hence, no participants’ names will be revealed even when submitting the information to sponsors or regulatory institutions. Both the participants’ records and the coding system will remain confidential and accessible only by the researchers. Consent forms will be provided in two copies to be signed by the participant and the researcher. A secure location containing a single folder will be dedicated to storing all the files.

The participant will not incur any personal expenses during the study; hence, all evaluations will be free. Additionally, no financial compensation or incentives will be given for participation. Any additional expenses will be covered by the research budget. Meanwhile, participation is completely voluntary, and there is no implication in the patient’s medical follow-up for nonparticipation. Upon signing the consent form, participants can withdraw from the study at any time, if desired, without any consequences related to their treatment or follow-up at the hospital. The anticipated research results may be published in a journal article or presented at meetings; nevertheless, the participants’ identities will remain confidential. Most importantly, this research poses no risk to the participants.

### Quality control measures

Several important methodological resources will be used to ensure quality data collection and minimise bias in the study. This includes the mixed-method study design, inclusion and exclusion criteria, random sampling technique and randomisation process. The principal researcher will collect all the data during the FGD, whereas additional enumerators will assist with the data collection during the quantitative phase. These enumerators will participate in distributing the questionnaire during the pilot test for them to get familiarised with the study instrument. Meanwhile, during the quantitative phase, the principal researcher will ensure that enumerators are following the survey protocol. Participants would be assisted while completing the questionnaire if necessary to minimise missing data. Another quality control measure is the principal researcher’s role by reviewing the completeness and quality of data in the returned questionnaires.

### Data analysis

For the qualitative phase, interview transcripts will be analysed using NVivo (Version 12). The software will be used to develop the main themes and sub-themes from the focus group discussion. Meanwhile, the quantitative data will be analysed using the IBM SPSS for Windows (version 25.0, Armonk, NY. IBM. Corp.). Cronbach alpha values will be computed from the reliability test to assess the reliability and to ensure the internal consistency of the questionnaire items. Cronbach alpha values of more than 0.7 will be accepted as reliable. Descriptive statistics will be used to summarise the data regarding participants’ socio-demographic characteristics, socio-cultural factors, health-seeking behaviours, and delayed presentation of TB.

The independent variable in this study is the delayed presentation of TB (measured in time: days and weeks), hence, the data will be subjected to normality tests using the level of kurtosis and skewness. Means and standard deviations will be presented for normally distributed data, whereas if the assumption of normality is not met, median and interquartile range will be presented. The cumulative incidence of TB will be estimated based on the number of new cases of TB divided by the total number of participants at risk. Determinants of delayed TB presentation will be analysed using multiple regression models. All parameter estimates will be computed at a 95% confidence interval, and statistical significance will be reported at *p* < 0.05. Lastly, a measurement model and structural modelling analysis will be performed using SmartPLS 3.0.

### Timeline

All participants for the qualitative phase have been recruited, and the study is expected to be completed by April 2022. Meanwhile, participants’ recruitment for the quantitative phase will commence in March 2022. The data analysis will be performed by August 2022, and the research article will be completed by October 2022.

## Conclusion

Based on the published literature, there is limited research on the social factors and their association with delayed presentation among TB patients. With the increasing prevalence of TB in Malaysia, our proposed research will be vital because it can provide an overview of the collective effects of social development and environmental factors on TB. It can also be used to evaluate if the social disadvantages outweigh the positive effects of overall growth in Malaysia. Furthermore, this study will shed insights into the social factors associated with the delayed presentation of TB so that targeted strategies can be implemented to reduce the local incidence of TB.
